# Supplementary material for: Splice-Junction-Based Mapping of Alternative Isoforms in the Human Proteome
Source: Cell Rep. Author manuscript; Available in PMC 2020 Jan 15. (PMC6961840; doi:10.1016/j.celrep.2019.11.026)
Supplement: 3 [file NIHMS1546469-supplement-3.zip › DF2/PXD0010154/Esophagus-16-Q01484-LIEVGPSGAQFLGPVIVEIPHFAALR.pdf]

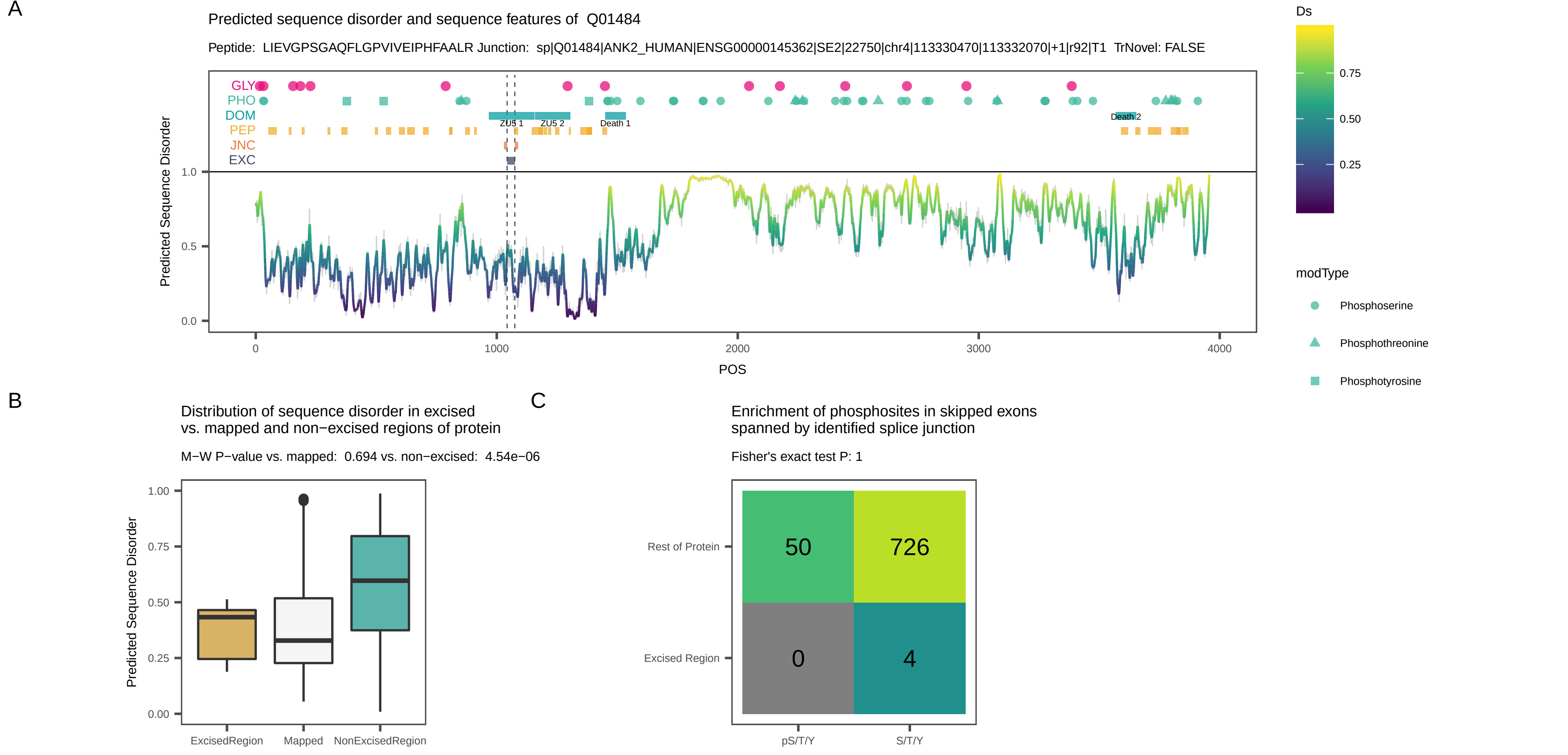
A
Predicted sequence disorder and sequence features of Q01484
Peptide: LIEVGPSGAQFLGPVIVEIPHFAALR Junction: sp|Q01484|ANK2_HUMAN|ENSG00000145362|SE2|22750|chr4|113330470|113332070|+1|r92|T1 TrNovel: FALSE
GLY
PHO
DOM
PEP
JNC
EXC
ZU5 1
ZU5 2
Death 1
Death 2
Predicted Sequence Disorder
1.0
0.5
0.0
0
1000
2000
3000
4000
POS
Ds
0.75
0.50
0.25
modType
Phosphoserine
Phosphothreonine
Phosphotyrosine
B
Distribution of sequence disorder in excised
vs. mapped and non−excised regions of protein
M−W P−value vs. mapped: 0.694 vs. non−excised: 4.54e−06
Predicted Sequence Disorder
1.00
0.75
0.50
0.25
0.00
ExcisedRegion
Mapped
NonExcisedRegion
C
Enrichment of phosphosites in skipped exons
spanned by identified splice junction
Fisher's exact test P: 1
Rest of Protein
Excised Region
50
726
0
4
pS/T/Y
S/T/Y
